# Cable Bacteria Activity Modulates Arsenic Release From Sediments in a Seasonally Hypoxic Marine Basin

**DOI:** 10.3389/fmicb.2022.907976

**Published:** 2022-07-13

**Authors:** Sebastiaan J. van de Velde, Laurine D. W. Burdorf, Silvia Hidalgo-Martinez, Martine Leermakers, Filip J. R. Meysman

**Affiliations:** ^1^Department of Geoscience, Environment and Society, Université Libre de Bruxelles, Brussels, Belgium; ^2^Operational Directorate Natural Environment, Royal Belgian Institute of Natural Sciences, Brussels, Belgium; ^3^Microbial Systems Technology, Department of Biology, University of Antwerp, Antwerp, Belgium; ^4^Analytical, Environmental and Geo-Chemistry, Department of Chemistry, Vrije Universiteit Brussel, Brussels, Belgium; ^5^Department of Biotechnology, Delft University of Technology, Delft, Netherlands

**Keywords:** electrogenic sulfur oxidation, marine sediments, long-distance electron transport, cable bacteria, arsenic, euxinia

## Abstract

Eutrophication and global change are increasing the occurrence of seasonal hypoxia (bottom-water oxygen concentration <63 μM) in coastal systems worldwide. In extreme cases, the bottom water can become completely anoxic, allowing sulfide to escape from the sediments and leading to the development of bottom-water euxinia. In seasonally hypoxic coastal basins, electrogenic sulfur oxidation by long, filamentous cable bacteria has been shown to stimulate the formation of an iron oxide layer near the sediment-water interface, while the bottom waters are oxygenated. Upon the development of bottom-water anoxia, this iron oxide “firewall” prevents the sedimentary release of sulfide. Iron oxides also act as an adsorption trap for elements such as arsenic. Arsenic is a toxic trace metal, and its release from sediments can have a negative impact on marine ecosystems. Yet, it is currently unknown how electrogenic sulfur oxidation impacts arsenic cycling in seasonally hypoxic basins. In this study, we presented results from a seasonal field study of an uncontaminated marine lake, complemented with a long-term sediment core incubation experiment, which reveals that cable bacteria have a strong impact on the arsenic cycle in a seasonally hypoxic system. Electrogenic sulfur oxidation significantly modulates the arsenic fluxes over a seasonal time scale by enriching arsenic in the iron oxide layer near the sediment-water interface in the oxic period and pulse-releasing arsenic during the anoxic period. Fluxes as large as 20 μmol m^−2^ day^−1^ were measured, which are comparable to As fluxes reported from highly contaminated sediments. Since cable bacteria are recognized as active components of the microbial community in seasonally hypoxic systems worldwide, this seasonal amplification of arsenic fluxes is likely a widespread phenomenon.

## Introduction

The global prevalence of seasonal hypoxia in coastal systems is increasing as a result of climate change and increased delivery of nutrients to the coastal ocean (Diaz and Rosenberg, 2008; Breitburg et al., 2018). Stratification coupled to high primary production leads to low oxygen concentrations in the bottom water of coastal water bodies in summer [“hypoxia,” defined as (O_2_) < 63 μM]. In some cases, the bottom water may become entirely devoid of oxygen (“anoxic”) and allows the escape of dissolved sulfide from the sediment, which can lead to the accumulation of sulfide in the bottom waters (named “euxinia”). At the same time, bottom-water oxygen limits the efflux of trace metals such as arsenic (As) from marine sediments by sustaining an iron oxide layer near the sediment-water interface (SWI), which acts as an efficient adsorption trap for trace metal(loid)s (Riedel et al., 1999). As the prevalence and frequency of hypoxia increase, trace metal(loid)s release from marine sediments is expected to increase (Banks et al., 2012). For a toxic trace metalloid like As, such an enhanced sedimentary efflux may harm the local marine ecosystem. Exposure to micromolar levels of As (both As^III^ and As^V^) is toxic to plants and animals, and the inorganic forms of As are also known carcinogens in humans (Sharma and Sohn, 2009).

In seasonally hypoxic Lake Grevelingen, the metabolic activity of cable bacteria during the oxic period in winter and spring limits sedimentary sulfide release during the anoxic period in summer by developing a “firewall” for dissolved sulfide (Burdorf et al., submitted; Seitaj et al., 2015). Cable bacteria are sulfur-oxidizing bacteria that perform electrogenic sulfur oxidation (e-SOx), which couples the oxidation of sulfide at depth to the reduction of oxygen near the SWI through electrical currents that run along the centimeter-long axis of these filamentous bacteria (Nielsen et al., 2010; Meysman et al., 2019). This centimeter-scale decoupling of redox half-reactions generates large characteristic pH excursions, inducing a broad and acidic zone at depth and a narrow and alkaline zone near the SWI (Nielsen and Risgaard-Petersen, 2014; Meysman et al., 2015; Meysman, 2017). The low pH at depth dissolves iron sulfides and releases dissolved iron in the pore water, of which part diffuses toward the oxic zone and precipitates as iron oxide (Risgaard-Petersen et al., 2012; Rao et al., 2016). This upward transport of reduced iron builds up a disproportionally large iron oxide layer during the oxic months, which subsequently provides an efficient cap (referred to as a “firewall”) for dissolved sulfide during the hypoxic and anoxic period (Burdorf et al., submitted; Seitaj et al., 2015). Active cable bacteria populations are also found in other seasonally hypoxic sites (Burdorf et al., 2017), which makes this possibly a global feature.

The As cycle in marine sediments is closely coupled to the iron cycle due to strong interactions between As and various iron minerals, such as iron (oxyhydr)oxides (further referred to as iron oxides) and iron-sulfide minerals (Mucci et al., 2000; Bostick and Fendorf, 2003; Chaillou et al., 2003; Wolthers et al., 2005; Couture et al., 2010). Under oxic bottom water conditions, As enters the sediment adsorbed onto iron oxides in settling particles. When these iron oxides are buried below the oxic zone, As is co-released with ferrous iron to the pore water during dissimilatory iron reduction (Edenborn et al., 1986; Peterson and Carpenter, 1986; Chaillou et al., 2003; Gao and Mucci, 2003). Consequently, the concentrations of dissolved As in the pore water are much higher than in the overlying water. The release of As from the sediment is generally small, as long as the water column is sufficiently oxygenated to sustain an iron oxide layer near the SWI. Only a limited fraction of As (0.02–0.9 μmol m^−2^ day^−1^) is generally able to diffuse through the oxic zone and into the overlying water (Peterson and Carpenter, 1986; Martin and Pedersen, 2002; Chaillou et al., 2003; Senn et al., 2007) because of the fast adsorption of As on the iron oxide oxides in the oxic zone (Couture et al., 2010). When oxygen concentrations in the overlying water decrease, the As efflux from sediments can become much higher (1.6–4.8 μmol m^−2^ day^−1^) (Riedel et al., 1999; Banks et al., 2012), as the iron oxide layer is no longer sustained.

The high affinity of As for iron oxides, together with the iron firewall mechanism induced by the cable bacteria, suggests that seasonally hypoxic systems may experience an amplified seasonal As cycle. Preliminary evidence indeed indicates that during the dissolution of iron sulfide minerals in the electro-active zone (the zone where e-SOx is active), As is released to the pore water (van de Velde et al., 2017). However, the evolution of the As cycle in coastal systems experiencing seasonal hypoxia remains poorly understood, and the impact of the iron firewall mechanism on As effluxes has not been examined. In this study, we presented *in-situ* pore-water and solid-phase data from three time points (i.e., March, May, and September 2015) in the seasonal cycle of Lake Grevelingen. These data are combined with results from a long-term core incubation experiment, where we monitored As release over several weeks after induction of anoxia. Our results demonstrated that the e-SOx metabolism of cable bacteria seasonally modulates the As release from the sediment, by limiting the As efflux in spring and stimulating the As efflux at the onset of hypoxia.

## Materials and Methods

### Field Site

Lake Grevelingen (N 51°44′, E 3°52′) is a saline coastal water body (area: 115 km^2^) in the Netherlands that is part of the former Rhine-Meuse-Scheldt estuary. Water exchange takes place via a sluice connection with the North Sea, and so a relative constant salinity (29–32) is maintained throughout the year. The study site was station “S1” in the Den Osse basin (23 m depth), which rapidly accumulates organic-rich sediments (~2 cm year^−1^) (Malkin et al., 2014). Seasonal oxygen depletion is a yearly occurring phenomenon at this site (Wetsteijn, 2011). In 2012, an extensive year-long sampling campaign has revealed the importance of e-SOx for sedimentary Fe, Mn, S, P, and Mo cycling in response to seasonally changing oxygen conditions (Seitaj et al., 2015; Sulu-Gambari et al., 2016a,b; Sulu-gambari et al., 2017). This dataset forms the background for this study.

### Water-Column and Sediment Core Sampling

Bottom water and sediment cores were collected on three different occasions (i.e., March, May, and August 2015). During each sampling campaign, water column depth profiles of temperature (T), salinity (S), and oxygen (O_2_) saturation were recorded with a CTD instrument (YSI 6600, YSI inc., USA). Bottom water was sampled with a 12 L NISKIN bottle, which was held stationary at 20 m depth for at least 10 min before retrieval. The bottom water was analyzed for O_2_, dissolved iron, and dissolved As. Afterwards, sediment cores were collected using a single core gravity corer (UWITEC, Austria) and transparent PVC core liners (inner diameter: 6 cm; length: 60 cm). Upon retrieval, sediment cores were carefully inspected and only cores with an apparent undisturbed SWI were kept for further analysis. Sediment cores were investigated with microsensor depth profiling at *in-situ* temperature in a temperature-controlled shipboard laboratory within 6 h from sampling. Subsequently, the sediment cores were transported back to a shore-based laboratory (NIOZ, Yerseke, The Netherlands) in a thermally insulated container for further analysis and experiments.

### Sediment Incubations

Whole-core sediment incubations were initiated the day after sediment core collection. Two cores were sectioned immediately before the incubation to determine the initial pore-water and solid-phase conditions. Three replicate cores were subsequently incubated, and the sediment levels of these cores were adjusted to have comparable volumes of overlying water (~15 cm). At the start of the incubation, about 75% of the overlying water in these cores was replaced with deoxygenated artificial seawater (salinity = 30); the cores were closed off with custom-built airtight polyoxymethylene lids equipped with a central stirrer and incubated in the dark in a temperature-controlled incubator (LT650 Elbanton, The Netherlands, 4°C—the *in-situ* temperature of the bottom water in March). Oxygen concentrations were continuously monitored (sampling frequency ~0.1 min^−1^) during the whole incubation using Oxygen Spot Sensors (OXPSP5; Pyroscience, Germany). Each week, the overlying water in the incubations was discretely sampled *via* two sampling ports in the lid, which allowed water sampling without O_2_ introduction. The water sample was analyzed for dissolved iron, dissolved sulfide, and dissolved As. To this end, glass syringes (Hamilton, USA) were connected to the sampling ports with tygon tubing. After sample collection, ~75% of the overlying water was removed and replaced with freshly prepared artificial seawater (salinity = 30), which was deoxygenated beforehand *via* nitrogen bubbling. The new artificial seawater was added carefully by placing a piece of bubble wrap on the top of the remaining water, which minimized disturbance of the sediment surface. Subsequently, the lid was replaced, and any remaining bubbles were removed by injecting anoxic water. Incubations continued for a few weeks after sulfide was detected in the overlying water (March: 23 weeks, May: 15 weeks, August: 7 weeks). In August, sulfide was detected after only 1 week of incubation, and thus the incubation time was much shorter for this month. At the end of the long-term incubation, duplicate cores were sectioned, and pore water and solid phase were analyzed (see below).

### Sediment and Pore Water Collection

Cores were sectioned for pore-water collection in an anaerobic glove box (N_2_ atmosphere with 3–5% H_2_; Coy lab products, USA). Cores were sectioned at 0.5 cm resolution from 0 to 6 cm depth and 1 cm resolution between 6 and 12 cm depth. Sediment slices were collected in 50 ml polypropylene centrifuge tubes (TPP, Switzerland) and centrifuged at 3,000 rpm for 10 min (Sigma 3–18KS, Sigma Laborzentrifugen GmbH, Germany). Subsequently, the centrifuge tubes were opened in the anaerobic glove box, and overlying pore water was transferred into suitable sample containers after filtration through 0.45 μm cellulose filters (Millex-HA filter, Merck Millipore, USA). Pore-water samples were analyzed for dissolved iron and dissolved As. To prevent oxidation, the solid phase that remained after centrifugation was freeze-dried and sealed in an airtight aluminum bag inside the anaerobic glove box and stored anaerobically for later solid-phase analysis.

### Microsensor Profiling

Microsensor profiling was carried out for dissolved H_2_S (100 μm tip diameter), O_2_ (50 μm tip), and pH (200 μm tip) using commercial micro-electrodes (Unisense A.S., Denmark). In each core, 2 replicate profiles were taken for each of the three parameters. Dissolved H_2_S was calibrated by making a five-point standard curve using Na_2_S standards, which were prepared on the day of analysis. For O_2_, a two-point calibration was made using air-saturated seawater (100% saturation) and the anoxic zone of the sediment (0% saturation). pH was calibrated by a 3-point calibration using standard NBS buffers (pH = 4, 7, and 10), followed by a salinity correction with Tris buffer (Dickson et al., 2007). Measurements of pH were performed using an external Ag/AgCl reference electrode, and values are reported on the total pH scale.

### Bottom-Water and Pore-Water Analysis

Water samples for dissolved Fe analysis were immediately stabilized with 50 μl of bidistilled HNO_3_ (65%) per ml of sample and preserved at 4°C. Before the analysis, samples were diluted 50 times with a standard matrix solution containing 35% artificial seawater and 2% HNO_3_ using 0.2 mg L^−1^ Ytterbium as an internal standard (Crompton, 1989). Samples were subsequently analyzed by inductively coupled plasma-optical emission spectroscopy (ICP-OES, ThermoFisher iCAP6500), precision was <2%. We will refer to concentrations determined by ICP-OES as dissolved Fe (dFe). Note, however, that the sample fraction obtained after filtration does not only consist of aqueous Fe^2+^ and dissolved complexes but potentially also of a fraction of colloidal and nanoparticulate iron (Raiswell and Canfield, 2012).

Samples for As analysis were stabilized and preserved identical as for dFe and were analyzed by high-resolution-inductively coupled plasma-mass spectroscopy (HR-ICP-MS, ThermoScientific Element 2) after 20× dilution with Milli-Q water. Indium (2.5 ppb) containing 2% HNO_3_ was injected simultaneously with the sample as an internal standard. Similar to iron, we will refer to As as dissolved As (dAs).

Samples for total free sulfide analysis [ΣH_2_S = (H_2_S) + (HS^−^)] were fixed with 100 μl ZnAc (10 %) per ml of sample and analyzed spectrophotometrically following the methylene blue method (Cline, 1969).

### Solid-Phase Analysis

To determine the solid-phase speciation of Fe and As within solid sediment phases, two extraction procedures were used (Supplementary Table 1). The first extraction uses an ascorbate solution that targets reactive Fe(III) phases, all Mn(III, IV) oxides and oxyhydroxides and associated trace metals (Kostka and Luther, 1994). The second extraction procedure employs a 1 M HCl solution, which extracts acid-volatile sulfides (AVSs), carbonate, amorphous Fe(III) phases, and elements from clay minerals (Kostka and Luther, 1994). In both procedures, 300 μg of dry sediment was extracted with 25 ml of solution (which was purged with N_2_ gas for 30 min before) for 24 h at ambient temperature under constant agitation. The addition of the solution was carried out inside an anaerobic chamber, to avoid oxidation artifacts. Afterwards, the solution was centrifuged and the supernatant was filtered (0.45 μm cellulose filters), diluted 20×, stabilized with 1% HNO_3_ and stored at 4°C. Analysis was carried out with HR-ICP-MS for Fe and As as described above for bottom- and pore-water analysis. Reproducibility (determined as the relative standard deviation of three replicates) for the ascorbate extraction was 3–7% for Fe and 2–5% for As. For the HCl extraction, reproducibility was 15–20 % for Fe and 20–30% for As.

Solid-phase inventories were calculated as follows:


(1) $$\begin{array}{l} INV=\int_{x_{up}}^{x_{down}}\left(1-\phi \right)\rho_{s}C_{solid}dx \end{array}$$

where C_solid_ is the solid-phase concentration (in mol g^−1^ dry weight), φ is the porosity, ρ_s_ is the density of the solid phase, and x_up_ and x_down_ are the depths over which the inventory was calculated. The porosity φ was determined from water content and solid-phase density measurements, accounting for the salt content of the pore water and averaged for the whole depth interval. The water content was determined as the volume of water removed when wet sediment samples were dried to constant weight at 60°C. Solid-phase density ρ_s_ at the field site was previously determined at 2.6 g cm^−3^ and assumed to be constant over the whole depth interval (Seitaj et al., 2017).

### Flux Calculations

The sediment efflux F_S_(t) of a given species S (dAs, dFe, or ΣH_2_S) was determined by calculating the solute inventory in the overlying water at the start and end of the weekly incubations. The inventory at the end simply amounts to V_OLW_[S]_end_, where V_OLW_ is the volume of the overlying water and [S]_end_ = [S]_t_ is the concentration of species S at time t. The inventory at the start of the incubation needs to account for the water replacement operation and becomes


(2) $$\begin{array}{l} V_{OLW}{\left[S\right]}_{start}=V_{repl}{\left[S\right]}_{repl}+\left(V_{OLW}-V_{repl}\right){\left[S\right]}_{t-1} \end{array}$$

where V_repl_ is the volume of the replaced overlying water, [S]_repl_ is the concentration of species S in the replacing seawater, and [S]_t−1_ is the concentration of S at the previous time point in the overlying water that remained. The efflux then can be calculated as follows:


(3) $$\begin{array}{l} F_{S}\left(t\right)=\frac{V_{OLW}{\left[S\right]}_{end}-V_{OLW}{\left[S\;\right]}_{start}}{A_{core}t} \end{array}$$

where Δt represents the exact duration of the incubation period (always ~7 days), and A_core_ is the surface area of the sediment (28.3 cm^2^).

The cumulative flux represents the total amount of species S that was released into the water column during the whole experiment and was calculated as follows:


(4) $$\begin{array}{l} \int_{0}^{t_{end}}F_{S}\left(t\right)dt=\sum F_{S}\left(t\right)\Delta t \end{array}$$

where t_end_ is the end of the experiment, and F_S_(t) is the flux at time t, as calculated by Equation (3).

Diffusive fluxes in the pore water are estimated using the Fick's first law (Fick, 1855) as follows:


(5) $$\begin{array}{l} J_{diff}=-\phi \frac{D_{0}\left(S,T\right)}{\theta^{2}}\frac{\delta C}{\delta x} \end{array}$$

where J_diff_ is the diffusive flux, C is the concentration in the pore water, x is the depth into the sediment, φ represents porosity, and θ^2^ = 1–2lnφ is the correction factor for sediment tortuosity (Boudreau, 1996). The molecular diffusion coefficient (D_0_) is calculated for measured salinity and temperature using the R package CRAN:marelac (Soetaert et al., 2010). The concentration gradient (δC/δx) was calculated by fitting a linear regression to the concentration profiles using the custom-made R script FLIPPER (https://github.com/sevdevel/FLIPPER) (van de Velde et al., 2022).

## Result and Discussion

### Sedimentary Biogeochemical Cycling in a Seasonally Hypoxic System

The combined O_2_, H_2_S, and pH microsensor profiles suggest a distinct seasonality in the geochemistry of Lake Grevelingen sediments, which are also reflected in the pore-water depth profiles of ferrous iron. This seasonality has been described in detail before and is due to drastic changes in the sedimentary microbial community and metabolism in response to the seasonal variation in bottom water oxygen concentrations in the seasonally hypoxic Lake Grevelingen (Seitaj et al., 2015; Sulu-Gambari et al., 2016a).

**Figure 1:**
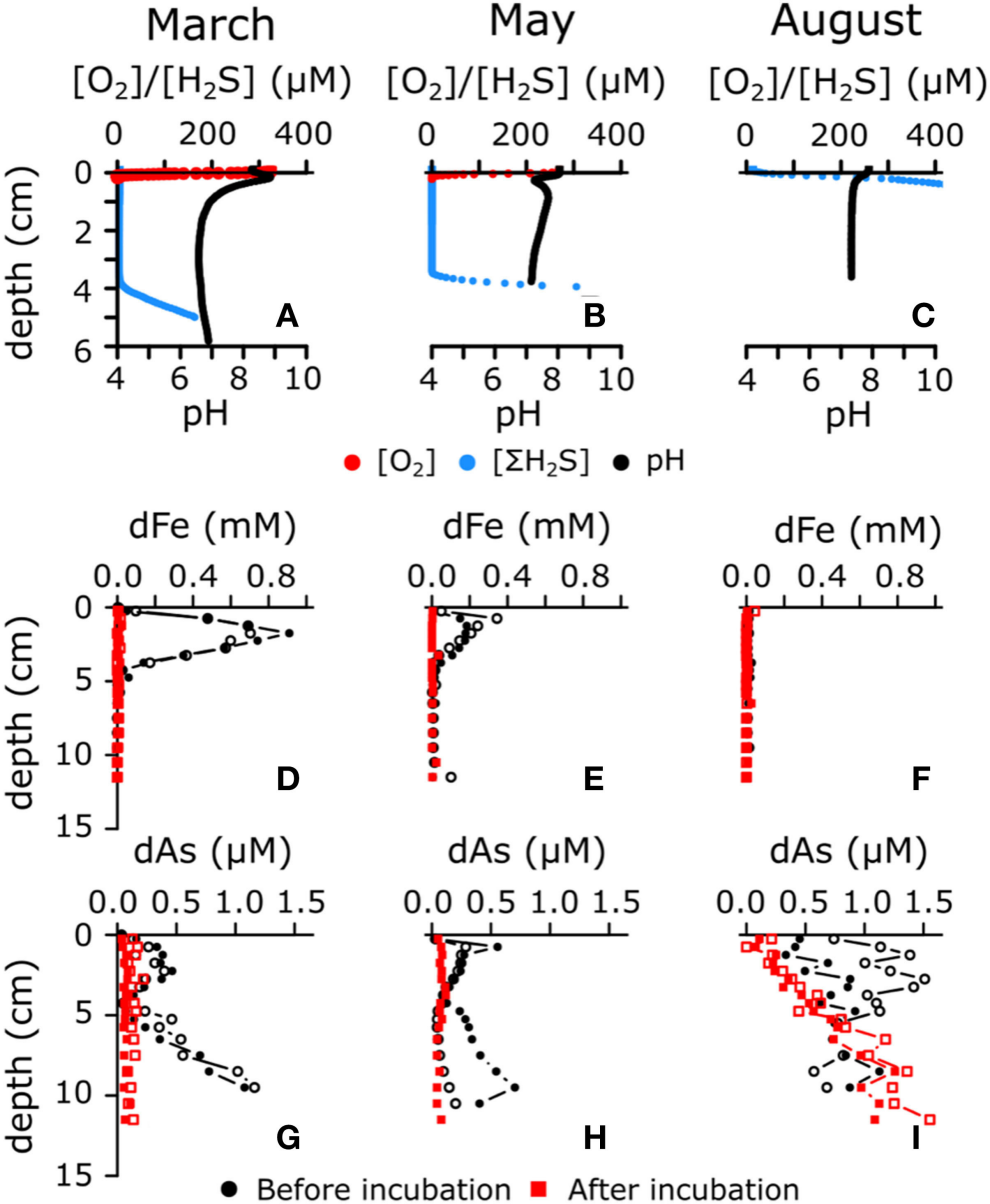
**(A–C)** Representative microsensor depth profiles of O_2_, H_2_S, and pH in three different seasons (i.e., March, May, and August 2015) in sediment of seasonally hypoxic Lake Grevelingen. All microsensor profile replicates are shown in Supplementary Figure 2. **(D–F)** Pore water depth profiles of dissolved iron in sediment upon core retrieval (denoted in black dots) and after prolonged incubation with anoxic overlying water (denoted in red squares). **(G–I)** Pore water profiles of dissolved arsenic before (denoted in black dots) and after (denoted in red squares) prolonged anoxic incubation. Open and filled symbols represent replicate cores.

In March 2015, the bottom water was fully oxygenated [(O_2_) = 329 μM], and microsensor profiling revealed the characteristic geochemical signature of electrogenic sulfur oxidation (e-SOx) by cable bacteria (Figure 1A; Nielsen et al., 2010; Meysman et al., 2015). A suboxic zone of ~40 mm deep was present, in which O_2_ and H_2_S remained below the detection limit (<1 μM). The pH depth profile showed a subsurface maximum (pH = 8.82 ± 0.03) near the oxygen penetration depth (OPD = 1.4 ± 0.3 mm) due to the proton consumption associated with cathodic reduction of oxygen


(6) $$\begin{array}{l} O_{2}+4e^{-}+4H^{+}\to 2H_{2}O \end{array}$$

while an acidic minimum (pH = 6.4 ± 0.1) was generated near the sulfide appearance depth (SAD = 41 ± 2 mm), resulting from proton production during anodic sulfide oxidation


(7) $$\begin{array}{l} H_{2}S+4H_{2}O\to SO_{4}^{2-}+8e^{-}+10H^{+} \end{array}$$

The low pH stimulated the dissolution of acid-sensitive minerals, most notably iron sulfide (Risgaard-Petersen et al., 2012; Rao et al., 2016; van de Velde et al., 2016), thus leading to strong dFe accumulation in the pore water (Figure 1D). The rate of FeS dissolution can be estimated by the sum of the diffusive fluxes just above and below the subsurface maximum dFe concentration (2.3 mmol m^−2^ day^−1^), which is comparable to other sediments with active e-SOx (Rao et al., 2016; van de Velde et al., 2016). Of the ferrous iron released in the pore water, 60% diffused toward the SWI, and the remaining 40% diffused downwards.

In May, bottom water [O_2_] was at 60% air saturation (175 μM), and the pH depth profiles revealed a subsurface minimum at the OPD (1.0 ± 0.3 mm), followed by a local maximum in the zone just underneath (Figure 1B). This specific type of pH depth profile has been associated with active cycling of iron between oxidized and reduced forms, where sediment mixing transports reduced forms of iron (FeS, adsorbed Fe^2+^) into the oxic zone and oxidized forms (FeOOH) downwards (Seitaj et al., 2015). This type of iron cycling requires an active form of sediment mixing such as bioturbation (van de Velde and Meysman, 2016; van de Velde et al., 2020), and indeed, we observed small polychaete tubes sticking out of the sediment (Burdorf et al., submitted). Evidence for burrowing fauna in May has also been found during a previous seasonal study of Lake Grevelingen (Seitaj et al., 2015, 2017). The proton production associated with the oxidation of reduced iron (FeS, Fe^2+^ or adsorbed Fe^2+^) and potential chemolithoautotrophy, led to a pH minimum in the oxic zone (pH = 7.10 ± 0.04)


(8) $$\begin{array}{l} 4Fe^{2+}+O_{2}+6H_{2}O\to 4FeOOH+8H^{+} \end{array}$$

whereas below 0.5 cm depth, the recovery of the pH to higher values (pH = 7.7 ± 0.2) is likely driven by the reduction of iron oxides (e.g., *via* sulfide-mediated iron reduction)


(9) $$\begin{array}{l} 8FeOOH+H_{2}S+14H^{+}\to 8Fe^{2+}+SO_{4}^{2-}+12H_{2}O \end{array}$$

Bioturbation generated a suboxic zone of 37 ± 7 mm, as the downmixing of iron oxides at depth prevented the accumulation of free sulfide (van de Velde and Meysman, 2016). Even though the redox pathways are likely more complex (e.g., by an intermediate cycle of manganese between oxygen and iron) (Aller, 1990; Sulu-Gambari et al., 2016a,b), iron cycling leads to an overall release of protons within the oxic surface layer and consumption of protons in the deeper suboxic zone (Jourabchi et al., 2005), as seen in the pH depth profile. The reductive dissolution of iron oxide also led to the release of dissolved iron in the pore water (Figure 1E), albeit at a lower rate than in March when e-SOx was active (Fe release rate = 1.4 mmol m^−2^ day^−1^, of which 78% diffuses toward the SWI).

The anoxic bottom water in August [(O_2_) < 1 μM] excluded e-SOx by cable bacteria (due to the lack of electron acceptors) and bioturbation (due to faunal mortality), and this is reflected in the microsensor depth profiles (Figure 1C). The pH decreased to a minimum of 7.3 ± 0.1 in the first 0.5 cm, after which it remained constant with depth, while ΣH_2_S started accumulating immediately at the SWI. This particular combination of pH and ΣH_2_S depth profiles is expected when sulfate reduction is the dominant pathway (Jourabchi et al., 2005):


(10) $$\begin{array}{l} 2CH_{2}O+SO_{4}^{2-}\to 2HCO_{3}^{-}+H_{2}S \end{array}$$

Strong sulfide production and the absence of bioturbation and e-SOx prevents the formation of a suboxic zone and inhibits the accumulation of dFe in the pore water through FeS precipitation (Figure 1F).

**Figure 2:**
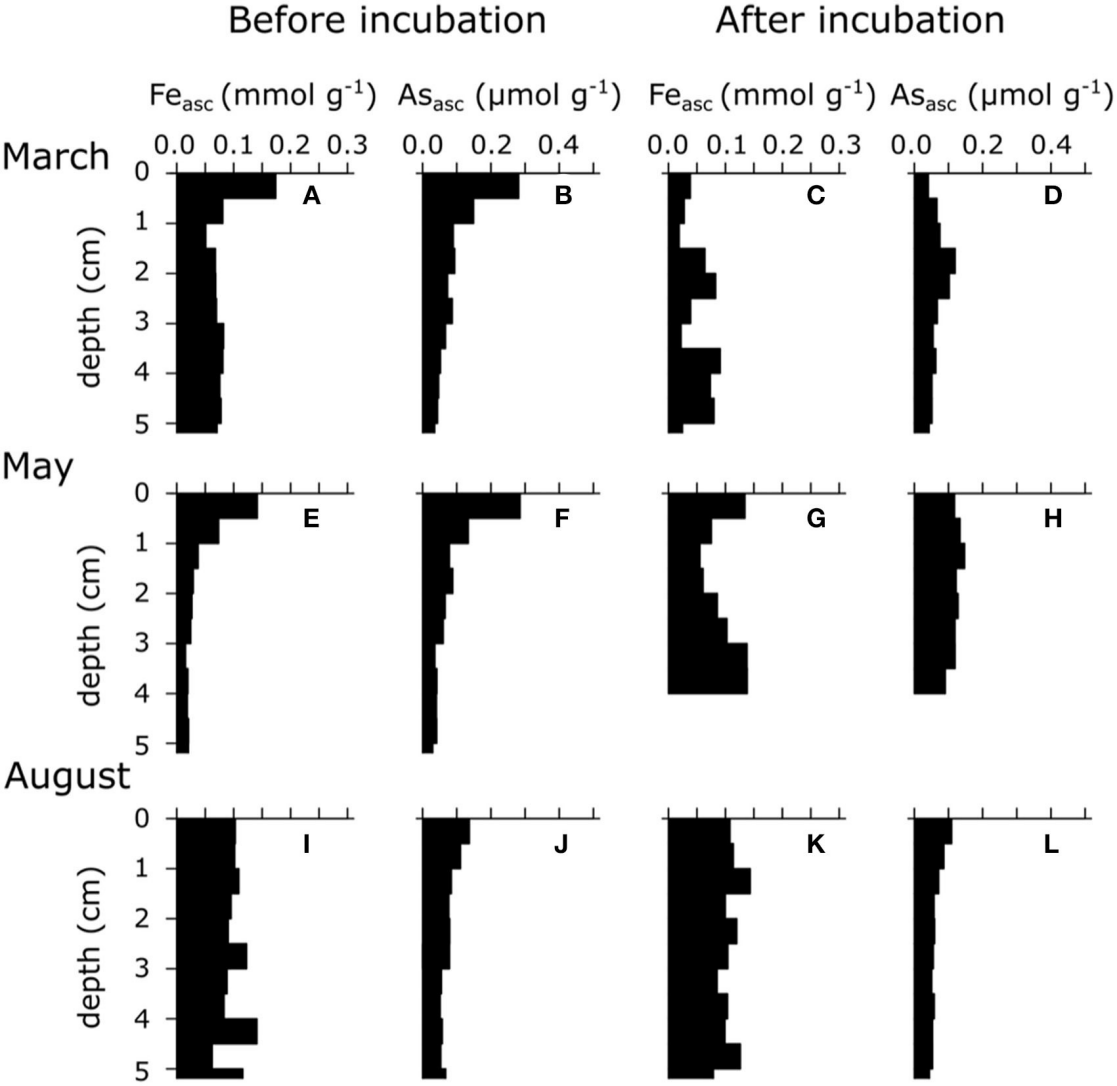
Ascorbate extractable Fe (Fe_asc_) and As (As_asc_) of three different seasons (i.e., March, May, and August 2015) in the sediment of seasonally hypoxic Lake Grevelingen. **(A,E,I)** Solid-phase ascorbate extractable iron depth profile upon core retrieval. **(B,F,J)** Solid-phase ascorbate extractable arsenic depth profile upon core retrieval. **(C,G,K)** Solid-phase ascorbate extractable iron depth profile after prolonged anoxic incubation. **(D,H,L)** Solid-phase ascorbate extractable arsenic depth profile after prolonged anoxic incubation. Results are expressed in μmol g^−1^ dry weight. Note the difference in x-axis for Fe_asc_ and As_asc_.

Overall, we found that iron and sulfur cycling in Lake Grevelingen shows a marked seasonality, alternating between dominance of e-SOx in March, bioturbation-driven metal cycling in May and sulfate reduction in August. This seasonal pattern is congruent with previous descriptions of the sediment biogeochemical cycling in Lake Grevelingen (Seitaj et al., 2015; Sulu-Gambari et al., 2016a) and shows that this strong seasonality in iron and sulfur cycling is likely a recurring phenomenon. The upward dFe fluxes were large in both March (1.4 mmol m^−2^ day^−1^) and May (1.1 mmol m^−2^ day^−1^), which suggests that substantial iron oxides were formed within the oxic zone, thus leading to the accumulation of iron oxides near the SWI (Figures 2A,E). However, there is an important difference between both periods. During e-SOx activity in March, iron is first transferred from reduced FeS to dissolved Fe in the pore water and subsequently precipitates as oxidized FeOOH. This is when a strong iron oxide “firewall” *sensu* Seitaj et al. (2015) is formed, and this engenders a one-way transport of iron from deeper layers to the oxic zone near the sediment surface. In May, when bioturbation is active, iron cycling is fundamentally different. FeOOH and FeS are cycled forth and back within the bioturbated zone, leading to a re-distribution of Fe over reduced and oxidized phases (Burdorf et al., submitted; Seitaj et al., 2015). As shown below, these different modes of iron cycling will differently control the sedimentary As cycle.

### Arsenic Cycling in a Seasonally Hypoxic Basin

In March 2015, when the water column was fully oxygenated, e-SOx was active in the sediment (Figure 1A and Section Sedimentary Biogeochemical Cycling in a Seasonally Hypoxic System), and a subsurface maximum of dAs was seen over the first 4 cm (coinciding with the suboxic zone; Figure 1G). In anoxic sediments, As adsorbs readily onto iron sulfides (Farquhar et al., 2002; Bostick and Fendorf, 2003; Wolthers et al., 2005). During e-SOx-driven dissolution of FeS, As was likely released into the pore water (production rate = 0.85 μmol m^−2^ day^−1^, of which 45% diffuses upwards), leading to accumulation of dAs in the pore water within the electro-active zone (Figure 1G; van de Velde et al., 2017). Alternatively, decreasing pH has been shown to lower the adsorption capacity of As onto iron sulfide minerals (Bostick and Fendorf, 2003; Wolthers et al., 2005). This could be an additional explanation for the As release seen in the electro-active zone. The observed dAs concentrations (0.2–0.5 μM) are comparable to those seen in bioturbated coastal sediments, where the As cycle is driven by iron oxide reduction (0.01–1.2 μM) (Edenborn et al., 1986; Peterson and Carpenter, 1986; Mucci et al., 2000, 2003; Chaillou et al., 2003). However, the dAs concentrations observed here are about four times lower than previously found in North-Sea sediments, where e-SOx was the dominant process (van de Velde et al., 2017). They are also lower than in deltaic sediments that sustain an active iron cycle (Sullivan and Aller, 1996) and contaminated lake sediments (Barrett et al., 2019; Leclerc et al., 2021), where pore water dAs can reach up to 2 μM (and sometimes up to 10 μM). Just below the zone of As release, the pore water showed a zone with strong dAs consumption, which corresponded to the interface between the ferruginous and the sulphidic pore water. This dAs removal is likely caused by intense FeS precipitation due to the supply of dissolved iron from the suboxic zone (Figure 1D). As this FeS is newly formed, and thus highly disordered, a lot of adsorption sites for As are created, which enables a strong sink for dAs (and leads to near-zero concentrations of dAs).

Part of the As diffusing upwards adsorbs onto iron oxides that are present near the SWI. Most of the upward diffusing As can be trapped in the oxic layer (0.3 μmol m^−2^ day^−1^), and only ~0.08 μmol m^−2^ day^−1^ can potentially diffuse into the water column (see As budget in Supporting Information). Trapping of As in the oxic zone leads to As accumulation in the iron oxide layer (up to 0.3 μmol As g^−1^) near the SWI (Figure 2B). Other studies have reported similar concentrations of particulate As in the top layer of coastal sediments (Mucci et al., 2000; Chaillou et al., 2003), higher concentrations are generally found in contaminated marine (0.4–0.7 μmol As g^−1^; Chaillou et al., 2003) and lake sediments (up to 15 μmol As g^−1^; Barrett et al., 2019; Leclerc et al., 2021).

Intriguingly, the pore water profiles suggest a strong upward flux of dAs coming from below the sampled depth of 12 cm. This upward flux was present in all cores of all three sampled months (black dots in Figures 1G–I) and disappeared after prolonged anoxic incubation (red squares in Figures 1G–I), which suggests that there is a deep and continuous source of dAs (below 15 cm depth). Note that the incubation time of the August cores was only 35 days, as compared with >100 days for the other 2 months, so there was less time for dAs to diffuse out of the sediment. This dAs source is possibly coming from crystalline iron oxides that were preserved and slowly dissolved afterwards or As associated with refractory organic matter (Chaillou et al., 2003). Alternatively, the As that was adsorbed on FeS could desorb due to the increase in dissolved sulfide with depth (Bostick and Fendorf, 2003), or As can be released when FeS becomes more ordered, which decreases the available adsorption sites. The affinity of As is a factor 5 lower for pyrite compared with FeS and so the gradual transformation of FeS into FeS_2_ in deeper sediments can also lead to As release (Bostick and Fendorf, 2003). Note that the incubated sediment cores only extend to 12 cm and are cut-off from this deeper As source that is present in natural sediments, which explains why dAs decreases at depth over time in the incubations.

In May 2015, the dAs peak was closer to the SWI (0.5 μM at 0.25 cm depth; Figure 1H), correlating with the highest As_asc_ concentration (0.3 μmol g^−1^; Figure 2F), which suggests that the As release was now mainly driven by dissimilatory iron reduction (Mucci et al., 2000; Chaillou et al., 2003; Couture et al., 2010). The production rate of As in May was almost two times higher than in March (1.52 μmol m^−2^ day^−1^ vs. 0.85 μmol m^−2^ day^−1^). In March, the main dAs source was the dissolution of FeS, which has a lower adsorption affinity for dAs than FeOOH. Accordingly, we conjectured that more As was co-released during iron reduction in May, thus explaining the higher release rates compared with those in March.

Below the zone of dAs release, the sink of dAs was less pronounced in May, suggesting that the trapping mechanism from early spring (strong FeS precipitation) was less potent due to lower pore water dFe concentrations (Figure 1E). The location of this sink was also higher, corresponding to the upwards migration of the sulfide horizon (Figure 1H). As_asc_ concentrations did not vary significantly between March and May (Figures 2B,F), suggesting that less dAs was lost from the sediment over that period, likely because the oxygen concentration in the overlying water was still sufficiently high to act as an efficient trap. Hence, sufficient iron oxides were still present (Figure 2E) to re-adsorb the dissolved As that was released during dissimilatory iron oxide reduction.

In summer, the whole sediment core was sulphidic (Figure 1C), and the concentration of As_asc_ dropped in the top sediment layer (<0.15 μmol g^−1^; Figure 2J). Dissolved As showed strong accumulation in the upper 5 cm (up to 1.5 μM at 2.5 cm depth; Figure 1I). Under high sulfide concentrations, As is known to adsorb onto FeS surfaces (Farquhar et al., 2002; Wolthers et al., 2005) or to form authigenic As sulfides (Mucci et al., 2000, 2003; O'Day et al., 2004), which would lead to low dAs concentrations (in contrast to what we see in this study). However, high sulfide concentrations also inhibit the adsorption capacity of As on iron sulfide mineral surfaces (Bostick and Fendorf, 2003). In the anoxic part of the sediment, iron sulfide minerals are abundant (Seitaj et al., 2015; Sulu-Gambari et al., 2016a; Burdorf et al., submitted), and a fraction of the dAs will be adsorbed onto these minerals (~0.05 μmol g^−1^ in the absence of dissolved sulfide; Supplementary Material). The upward migrating ΣH_2_S horizon can then lead to a transient increase of dAs by a relatively small decrease in the adsorption capacity of As on iron sulfides (a decrease of ~10% in adsorption capacity can lead to pore water dAs concentrations of >1 μM; Supplementary Material).

**Figure 3:**
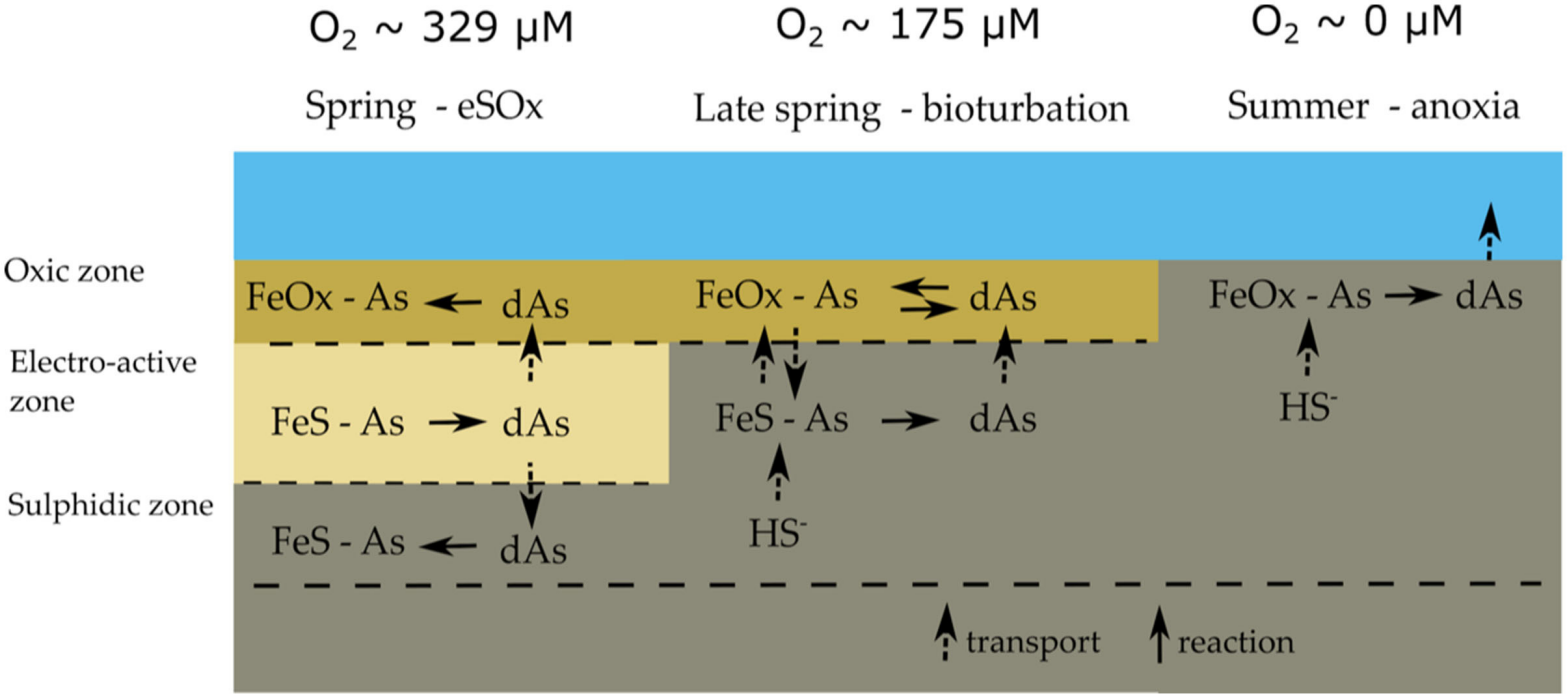
Conceptual model of the sedimentary arsenic cycle in seasonally hypoxic Lake Grevelingen; typical bottom water oxygen concentrations are indicated above. The dominant mode of geochemical cycling alternates between electrogenic sulfur oxidation in spring, bioturbation in late spring and anoxia in summer. See main text for details.

The combination of solid-phase and pore-water data suggest a strong link between the As cycle and the iron firewall mechanism induced by electrogenic sulfur oxidation (e-SOx), which can be summarized in a conceptual scheme (Figure 3). During winter and spring, e-SOx drives the dissolution of FeS and stimulates the formation of an iron oxide layer near the SWI (Seitaj et al., 2015; Sulu-Gambari et al., 2016a,b). Simultaneously, dAs is transferred from FeS in the deeper sediment layers to FeOOH near the SWI, leading to an enrichment of As in the iron oxide layer (Figures 2B, 3). In late spring, the sediment geochemistry changes to a bioturbation-driven iron cycle, which decreases the magnitude of the iron trap by the down-mixing of FeOOH and its subsequent conversion into FeS phases (Burdorf et al., submitted; Seitaj et al., 2015). During this downward mixing, As is released from the reduction of iron oxides and re-adsorbs on the iron oxides still present in the oxic zone, which leads to a constant concentration of dAs concentration in the iron oxide layer (Figures 2B, 3). However, after spring, when oxygen concentrations in the bottom water gradually decrease to eventually result in anoxia, the sulfide-mediated dissolution of the iron oxides will release all the adsorbed As into the overlying water column (Figures 2J, 3).

### Benthic-Pelagic Coupling

**Figure 4:**
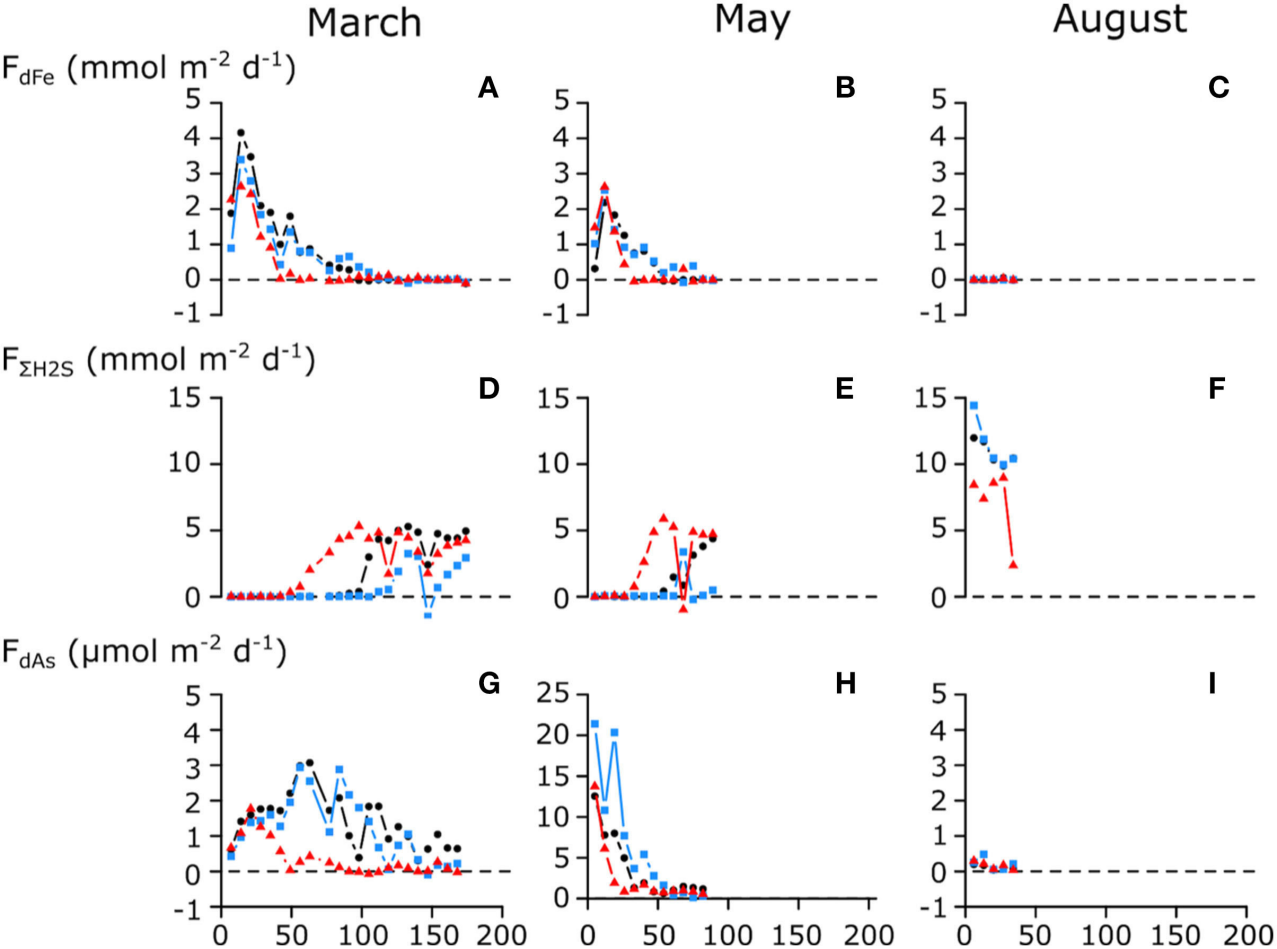
Measured benthic fluxes of **(A–C)** dissolved Fe (dFe); **(D–F)** total free sulfide (ΣH_2_S); and **(G–I)** dissolved As (dAs) during the long-term anoxic incubations of cores from seasonally hypoxic Lake Grevelingen. Incubations were stopped when ΣH_2_S was detected in the overlying water of all three cores. Cores were collected during different seasons; **(A,D,G)** March 2015; **(B,E,H)** May 2015; and **(C,F,I)** August 2015. Different colors indicate replicate cores. Note the difference in y-axis for the dissolved arsenic flux on **(H)**.

The fluxes of As, Fe, and ΣH_2_S that were recorded weekly during the sediment incubation experiment fully support the above model of the seasonal As cycle in Lake Grevelingen (Figure 3). In March, dFe was immediately released upon the introduction of anoxia with a maximum flux of 4 mmol Fe m^−2^ day^−1^ in the first 2 weeks, while in contrast, no detectable ΣH_2_S efflux was noted for 50–100 days (Figures 4A,D). As was co-released with iron (Figure 4G), though with a delay, and its maximum flux was only achieved after 50 days (~3 μmol m^−2^ day^−1^). This suggests that in the early stages of the incubations, some of the As was re-adsorbed onto the iron oxide layer that was still present. When the sulfide efflux effectively started after 100 days, As still escaped the sediment, though fluxes gradually diminished (~1.5 μmol m^−2^ day^−1^). This suggests that authigenic As sulfides do not form fast enough to trap all the dAs, and hence, they do not form an efficient sink for As. The measured As fluxes are typical for sediments underlying anoxic bottom waters (1.6–4.8 μmol m^−2^ day^−1^) (Riedel et al., 1999; Banks et al., 2012).

In May, dFe was also immediately released and quickly increased to a maximum flux (2–3 mmol m^−2^ day^−1^) that was about half that of March. Due to the presence of iron oxides in the top layer (Figure 2E), the ΣH_2_S release was also delayed, but now only for 30–50 days (Figures 4B,E), revealing that the strength of the iron firewall had diminished (Burdorf et al., submitted). In contrast, the maximum dAs efflux (15–20 μmol m^−2^ day^−1^) was much higher compared with March, and no delayed response was observed (Figure 4H). In August, no detectable dFe or dAs fluxes were measured, and ΣH_2_S was immediately released, indicating that the iron oxide layer was exhausted (Figures 4C,F,I).

The iron oxide trapping capacity was smaller in May compared with March, likely because part of the FeOOH was transported down by bio-mixing and converted into FeS (Burdorf et al., submitted; Seitaj et al., 2015). This is confirmed by the cumulative Fe flux, which was two times lower in May (69–134 mmol m^−2^) compared with March (52–60 mmol m^−2^) (Table 1). Cumulative As fluxes strongly correlated with cumulative Fe fluxes in both March and May (Supplementary Figure 3). In March, the As/Fe ratio of the cumulative flux was 0.003, while in May, it was 5 times higher (0.014), indicating that ~5 times more As (relative to Fe) was released in May. This suggests that during the initial dissolution stages of the iron oxide layer, a fraction of the As re-adsorbed on the iron oxide layer (consistent with the low As fluxes in the first weeks of the March incubation). In May, the change in solid-phase inventory before and after the experiment, estimated *via* the ascorbate extraction (0.44 mmol As m^−2^), agreed well with the cumulative As flux (0.32 ± 0.15 mmol As m^−2^; Table 1), indicating that only a small amount of As co-precipitated with sulfide or was adsorbed onto existing iron sulfides. In March, however, there was a larger difference (0.84 vs. 0.17 ± 0.09 mmol m^−2^; Table 1), which suggests that more As was retained in the sediment (Figures 4G,H). Possibly, the constant re-adsorption of As onto the iron oxides retained As in the sediment, which exposed it to sulfide in the pore water for a prolonged time. This could allow the formation and precipitation of authigenic As sulfides in March, which would not have been possible when As immediately diffused into the water column in May.

**Table 1:** Total release of dissolved arsenic (dAs), dissolved iron, and sulfide from the sediment over the course of the incubations (cumulative flux), compared with the inventory change of the ascorbate extraction.

		**Cumulative flux (mmol m** ^**−2**^ **)**			**Solid-phase inventory change (mmol m** ^**−2**^ **) in the upper 1 cm**		
		**March**	**May**	**August**	**March**	**May**	**August**
dAs	Core 1	0.24	0.28	0.004	0.841	0.436	0.135
	Core 2	0.20	0.48	0.005			
	Core 3	0.06	0.18	0.007			
dFe	Core 1	134	52	0.33	491	14.1	−45.4
	Core 2	112	60	0			
	Core 3	69	40	0.21			
ΣH_2_S	Core 1	335	100	368			
	Core 2	106	29	385			
	Core 3	450	23	242			

*Positive values indicate net loss; negative values indicate net gain. All units are in mmol m^−2^. Solid-phase inventory changes were calculated from ascorbate extractable Fe and As concentrations before and after incubation (Figure 2)*.

### Impact of the Cable Bacteria Induced Enhanced Seasonality

The iron oxide “firewall” mechanism induced by cable bacteria activity enhances the seasonality of the sedimentary As cycle and its release to the water column. During the oxic season, As is released from the dissolution of iron sulfides and accumulates in the iron oxide layer that is formed at the SWI (Figure 3). Subsequently, the sulfide-mediated dissolution of the iron oxide layer during the anoxic period leads to a pulse release, with high benthic As fluxes (up to 20 μmol m^−2^ day^−1^; Figure 4H). This is comparable to As fluxes estimated from pore-water gradients in contaminated lakes with an order of magnitude higher than solid-phase As concentrations (Barrett et al., 2019) and ~3–4 times higher than previously reported from anoxic sediment incubations (Riedel et al., 1999; Banks et al., 2012). When oxic conditions are subsequently re-stored in autumn and winter, the re-establishment of e-SOx restarts the formation of a new iron oxide layer and the trapping of As restarts. A seasonal study of Lake Grevelingen has shown that this also similarly affects phosphate and molybdenum (Sulu-Gambari et al., 2016b; Sulu-gambari et al., 2017), and it is not unlikely that this mechanism is also active for other elements that have a strong interaction with iron oxides (e.g., silicate and cobalt).

The high As efflux potentially leads to an increased As inventory in the water column. Whether this leads to toxic concentrations depends on the bottom water volume over which the released As is diluted. Water-column profiling (Hagens et al., 2015) shows that the mixed bottom layer in Lake Grevelingen extends over ~15 m. Using the minimum and maximum cumulative As flux (Table 1), this leads to a possible range of As concentrations of 4–32 nM. As a reference, the US EPA guideline for acute exposure of marine animals to arsenite (the reduced form of As, which is stable under anoxic conditions) is 920 nM (Neff, 1997), which suggests that the As release from the sediment of Lake Grevelingen is likely not accumulating up to toxic levels. Yet, in sediment with a low solid-phase As content, we measured high fluxes during the anoxic incubation. In contaminated marine sediments, where sedimentary As concentrations are elevated, even higher fluxes are expected, and the seasonal amplification induced by cable bacteria might pose a previously unknown environmental risk.

## Data Availability Statement

The raw data supporting the conclusions of this article will be made available by the authors, without undue reservation.

## Author Contributions

SV conceived the hypothesis. FM designed the experiment. LB and SH-M performed the field sampling and incubation experiment. SV and LB did the sediment extractions. ML analyzed the arsenic samples. SV and FM wrote the manuscript. All authors contributed to manuscript revision, read, and approved the submitted version.

## Funding

This research was supported by the Fonds Wetenschappelijk Onderzoek (Grant No. G038819N), the Universiteit Antwerpen (Grant No. TOPBOF), the Netherlands Organization for Scientific Research (Grant No. 016.VICI.170.072), and the Belgian Federal Science Policy Office (Grant No. FED-tWIN2019-prf-008). The HR-ICP-MS instrument was financed by the HERCULES Foundation (Code: UABR/11/010). The experimental work for this manuscript was carried out when SV was supported by a Ph.D. fellowship of the Research Foundation Flanders. SV was currently supported by the Belgian Federal Science Policy Office (Grant No. FED-tWIN2019-prf-008).

## Conflict of Interest

The authors declare that the research was conducted in the absence of any commercial or financial relationships that could be construed as a potential conflict of interest.

## Publisher's Note

All claims expressed in this article are solely those of the authors and do not necessarily represent those of their affiliated organizations, or those of the publisher, the editors and the reviewers. Any product that may be evaluated in this article, or claim that may be made by its manufacturer, is not guaranteed or endorsed by the publisher.
