# Supplementary material for: Cable Bacteria Activity Modulates Arsenic Release From Sediments in a Seasonally Hypoxic Marine Basin
Source: Front Microbiol. 2022 Jul 13;13:907976. doi: 10.3389/fmicb.2022.907976 (PMC9329047; doi:10.3389/fmicb.2022.907976)
Supplement: Supplementary file 1 [file Data_Sheet_1.PDF]

Supplementary Material

1 Supplementary Data

Arsenic adsorption on iron oxides:

In marine sediments, concentrations of dissolved arsenic are controlled by adsorption reactions on mineral surfaces, of which interactions with iron oxides (in the oxic layer near the sediment-water interface) and iron sulphides (in the anoxic, deeper, sediment layers) are the most important (Farquhar et al., 2002; Giménez et al., 2007; O'Day et al., 2004; Wolthers et al., 2005). Whether the adsorption process is effective (i.e. all dissolved species are adsorbed during transport through the layer of minerals, e.g., the iron oxide rich zone), depends on the (i) kinetic rate of adsorption and (ii) the rate of transport (molecular diffusion). Fast diffusional transport near the sediment-water interface can lead to benthic arsenic fluxes under oxic conditions (Martin and Pedersen, 2002; Senn et al., 2007).

The potential rate of adsorption (R_{ads}) on iron oxides can be estimated from the dissolved arsenic concentration, the iron oxide concentration and a kinetic constant k_{ads}

$$R_{ads} = k_{ads}[As]_{aq}\,\rho_{sed}(1-\phi)[Fe-ox]_{sp} \qquad [1]$$

Couture et al. (2010) have estimated a field value for k_{ads} (1.0 ± 0.4 10^{-2} cm^3 pore water μmol^{-1} d^{-1}), employing this estimate, and an arsenic concentration of 0.1 µM, an iron oxide concentration of 100 µmol g^{-1} (Figure 2a in main text), a solid phase density (ρ_{sed}) of 2.6 g cm^{-3} and a porosity of 0.8, we calculate an adsorption rate of 50 ± 20 µmol m^{-3} d^{-1}. Assuming that the iron oxide layer is about 0.5 cm thick (this is the resolution of our slicing at the SWI, and we used an average iron oxide concentration for that layer), we estimate that 0.3 ± 0.1 µmol As m^{-2} d^{-1} can be adsorbed during diffusion through the oxic layer.

Arsenic adsorption on iron sulphides and inhibition by free sulphide :

In anoxic sediment, arsenic can adsorb on iron sulphide mineral surfaces (FeS and FeS_2) (Bostick and Fendorf, 2003; Wolthers et al., 2005). Partitioning coefficients (P) of adsorbed arsenic ($[As]_{ad}$, the adsorbed concentration on 1 g of FeS) versus dissolved arsenic ($[As]_{aq}$), are expressed as

$$P = \frac{[As]_{ad}}{[As]_{aq}} \qquad [2]$$

For As(III) and As(V), values for P are 2 L g^{-1} FeS and 9 L g^{-1} FeS resp., indicating a stronger adsorption capacity for oxidised arsenic (Wolthers et al., 2005) (these values are determined in low-sulphide environment). With a dissolved arsenic concentration ($[As]_{aq}$) of 0.5 µM, this would lead to an adsorbed arsenic concentration between 1 and 4.5 µmol g^{-1} FeS. We can correct this range for the amount of FeS per g of sediment (200 µmol g^{-1}, or 0.02 g FeS g^{-1} of sediment, Supplementary Figure

1; Burdorf et al., submitted), and come to 0.02 – 0.08 µmol As g^{-1} sediment. We did not determine adsorbed arsenic directly, but we can consider $[As]_{asc}$ as a proxy for adsorbed and oxide-associated arsenic. Below the oxic zone (where oxides will be largely absent), $[As]_{asc}$ will then mostly consist of loosely adsorbed arsenic (Kostka and Luther III, 1994). The theoretical adsorbed arsenic value is close to the $[As]_{asc}$ between 1 and 5 cm (below the oxic zone) (~0.1 µmol g-1; Figure 2 in the main text), the difference might be due to arsenic associated with more refractory iron oxides.

High dissolved sulphide concentrations inhibit arsenic adsorption on sulphide mineral surfaces (Bostick and Fendorf, 2003) (and thus essentially decrease the P value), which suggests that the high dissolved arsenic concentrations in August in the upper 5 cm could be explained by a decrease of the adsorption capacity of arsenic (due to the upward migrating sulphide horizon). If the increase in dissolved sulphide decreased the P value by a couple of percent (~10 % for the lower end and ~3 % for the upper end), the $[As]_{ad}$ concentration for the same dissolved arsenic concentration (0.5 µM) would decrease with ~0.002 µmol g^{-1}, which corresponds with a pore-water concentration of 1 µM (Figure 1f in the main text, using a conversion factor of $\phi / \rho(1-\phi)$, where ϕ is the porosity and ρ the solid phase density). In laboratory experiments, the addition of 100 µM of dissolved sulphide has been shown to decrease the amount of adsorbed As(III) (for the same dissolved arsenic concentration) on pyrite by a factor 2 (Bostick and Fendorf, 2003), and thus a decrease of 10 % in adsorption partitioning coefficient for an increase of dissolved sulphide from 0 µM to > 400 µM (Figure 1b,c in the main text) is not unrealistic. It is thus not unlikely that a transient change in adsorption could lead to the high dissolved arsenic concentrations in August. The transient nature of the profiles could also explain the high variability (as compared to the other months, were the profiles were much smoother and comparable between replicates).

<u>References</u>

Bostick, B. C., and Fendorf, S. (2003). Arsenite sorption on troilite (FeS) and pyrite (FeS2). *Geochim. Cosmochim. Acta* 67, 909–921. doi:10.1016/S0016-7037(02)01170-5.

Couture, R. M., Gobeil, C., and Tessier, A. (2010). Arsenic, iron and sulfur co-diagenesis in lake sediments. *Geochim. Cosmochim. Acta* 74, 1238–1255. doi:10.1016/j.gca.2009.11.028.

Farquhar, M. L., Charnock, J. M., Livens, F. R., and Vaughan, D. J. (2002). Mechanisms of arsenic uptake from aqueous solution by interaction with goethite, lepidocrocite, mackinawite, and pyrite: An X-ray absorption spectroscopy study. *Environ. Sci. Technol.* 36, 1757–1762.

Giménez, J., Martínez, M., de Pablo, J., Rovira, M., and Duro, L. (2007). Arsenic sorption onto natural hematite , magnetite , and goethite. *J. Hazard. Mater.* 141, 575–580. doi:10.1016/j.jhazmat.2006.07.020.

Kostka, J. E., and Luther III, G. W. (1994). Partitioning and speciation of solid phase iron in saltmarsh sediments. *Geochim. Cosmochim. Acta* 58, 1701–1710. doi:10.1016/0016-7037(94)90531-2.

Martin, A. J., and Pedersen, T. F. (2002). Seasonal and Interannual Mobility of Arsenic in a Lake Impacted by Metal Mining. *Environ. Sci. Technol.* 36, 1516–1523. doi:10.1021/es0108537.

O’Day, P. A., Vlassopoulos, D., Root, R., and Rivera, N. (2004). The influence of sulfur and iron on dissolved arsenic concentrations in the shallow subsurface under changing redox conditions. *Proc. Natl. Acad. Sci. U. S. A.* 101, 13703–13708. doi:10.1073/pnas.0402775101.

Senn, D. B., Gawel, J. E., Jay, J. A., Hemond, H. F., and Durant, J. L. (2007). Long-term fate of a pulse arsenic input to a eutrophic lake. *Environ. Sci. Technol.* 41, 3062–3068. doi:10.1021/es062444m.

Wolthers, M., Charlet, L., van Der Weijden, C. H., van der Linde, P. R., and Rickard, D. (2005). Arsenic mobility in the ambient sulfidic environment: Sorption of arsenic(V) and arsenic(III) onto disordered mackinawite. *Geochim. Cosmochim. Acta* 69, 3483–3492. doi:10.1016/j.gca.2005.03.003.

Supplementary Figures and Tables

1.1 Supplementary Tables

**Ascorbate solution**	Total volume:	1L
Reagent	*mass (g)*	
Sodium bicarbonate ($NaHCO_3$)	50	
Trisodium citrate ($Na_3C_6H_5O_7$)	50	
ascorbic acid ($C_6H_8O_6$)	20	
**HCl solution**	Total volume:	1L
Reagent	*Volume(mL)*	
Hydrochloric acid (37%) (HCl)	83	

Supplementary Table 1: Recipes for extractions reagents used for the extraction of arsenic and iron from the solid phase.

1.2 Supplementary Figures

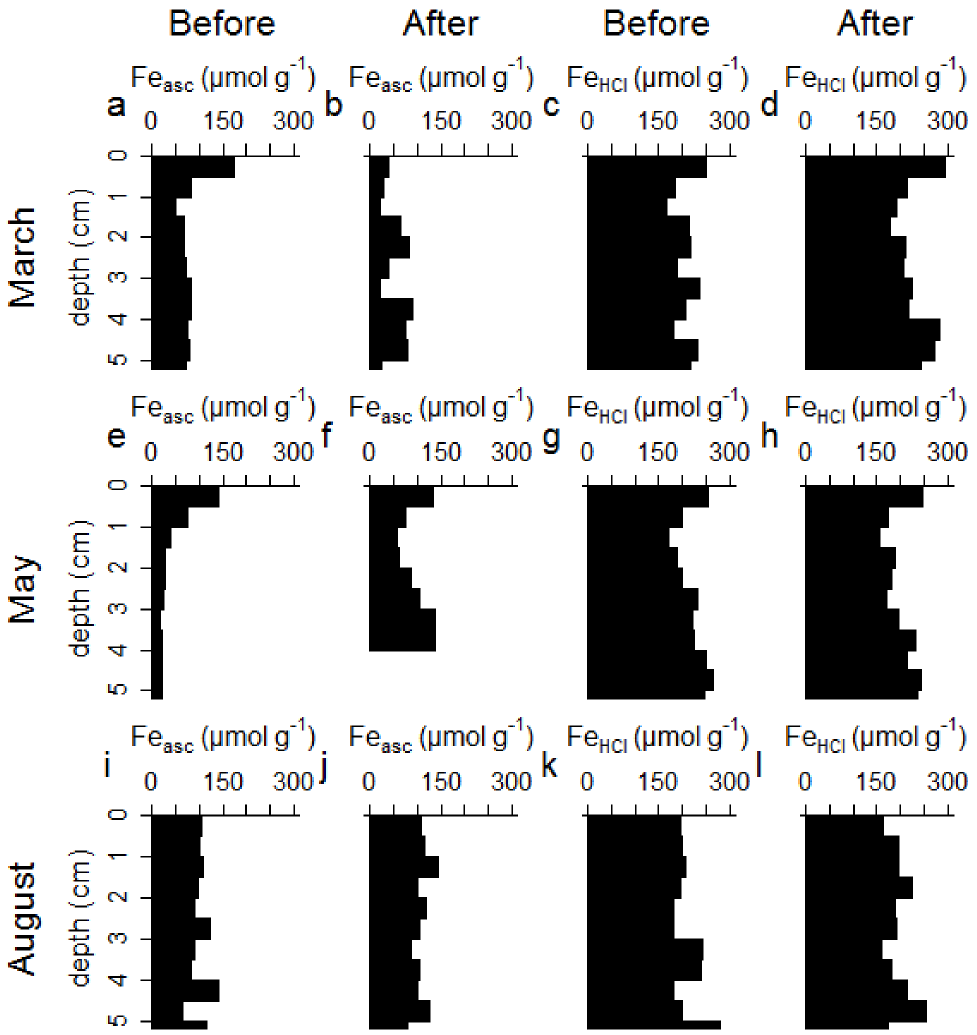

Supplementary Figure 1. Ascorbate extractable Fe (Fe_{asc}) and HCl extractable iron (Fe_{HCl}) of three different seasons (March, May, August 2015) in the sediments of seasonally hypoxic Lake Grevelingen. (a), (e), (i) Solid phase ascorbate extractable iron depth profile upon core retrieval, (b), (f), (j) solid phase ascorbate extractable iron depth profile after prolonged anoxic incubation. (c), (g), (k) Solid phase HCl extractable iron depth profile upon core retrieval, (d), (h), (l) solid phase HCl extractable iron depth profile after prolonged anoxic incubation. Results are expressed in µmol g^{-1} dry weight.

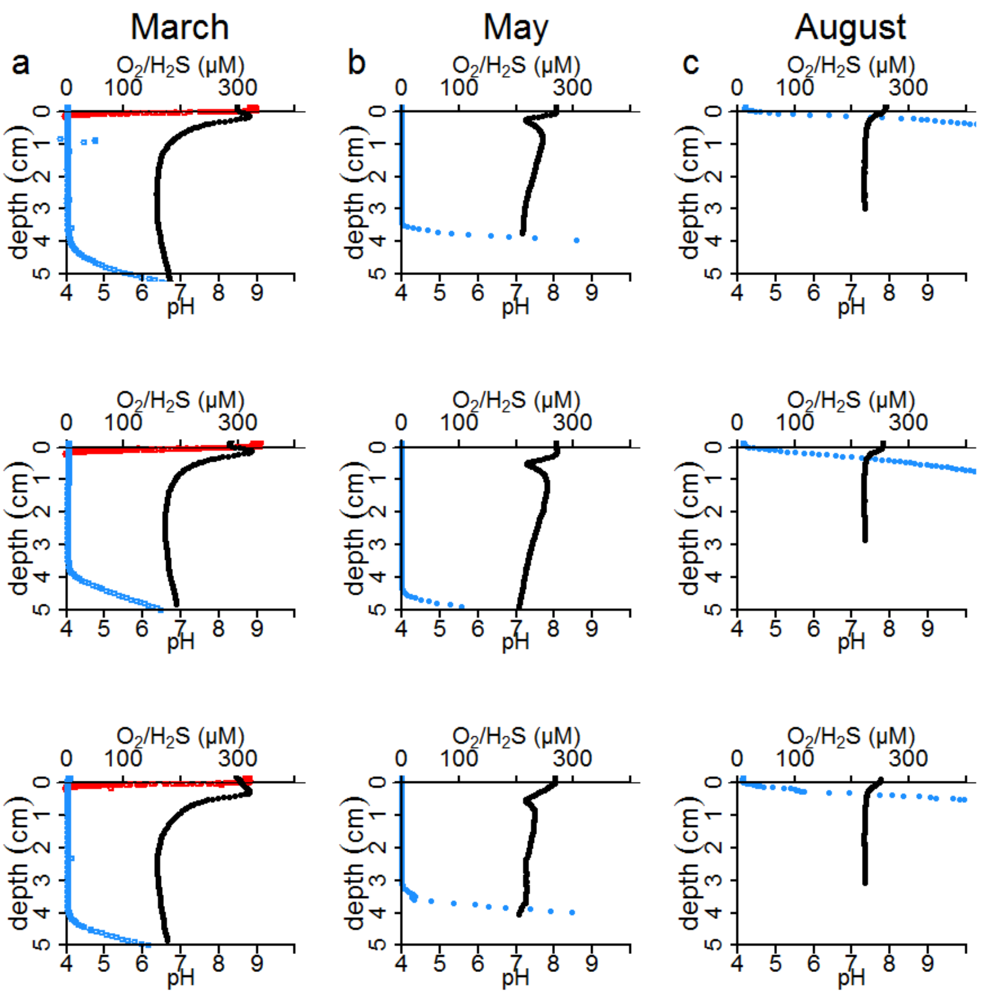

Supplementary Figure 2. Microsensor depth profiles of O_2, H_2S and pH from three replicate cores of the sediments of seasonally hypoxic Lake Grevelingen, retrieved in three different seasons; (a) March, (b) May and (c) August 2015.

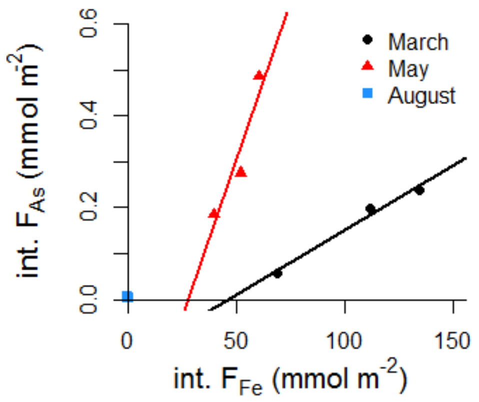

Supplementary Figure 3. Correlation between the cumulative fluxes of iron and arsenic for the individual cores of the incubation of Lake Grevelingen sediment, retrieved in three different seasons (March, May and August 2015).
